# Conformational Landscape of the *PRKACA-DNAJB1* Chimeric Kinase, the Driver for Fibrolamellar Hepatocellular Carcinoma

**DOI:** 10.1038/s41598-017-18956-w

**Published:** 2018-01-15

**Authors:** Michael D. Tomasini, Yingjie Wang, Adak Karamafrooz, Geoffrey Li, Thijs Beuming, Jiali Gao, Susan S. Taylor, Gianluigi Veglia, Sanford M. Simon

**Affiliations:** 1 Laboratory of Cellular Biophysics, The Rockefeller University, 1230 York Avenue, New York, NY 10065 USA; 2 Department of Chemistry, University of Minnesota, Minneapolis, MN 55455 USA; 3 Department of Biochemistry, Molecular Biology, and Biophysics. University of Minnesota, Minneapolis, MN 55455 USA; 4 Department of Pharmacology, University of California, San Diego, CA 92093 USA; 5 Schrödinger Inc., 120 West 45th Street, New York, NY 10036 USA; 6 Theoretical Chemistry Institute, Jilin University, Changchun, Jilin Province 130028 People’s Republic of China; 7 Department of Chemistry and Biochemistry, University of California, San Diego, CA 92093 USA

## Abstract

In fibrolamellar hepatocellular carcinoma a single genetic deletion results in the fusion of the first exon of the heat shock protein 40, *DNAJB1*, which encodes the J domain, with exons 2–10 of the catalytic subunit of protein kinase A, *PRKACA*. This produces an enzymatically active chimeric protein J-PKAcα. We used molecular dynamics simulations and NMR to analyze the conformational landscape of native and chimeric kinase, and found an ensemble of conformations. These ranged from having the J-domain tucked under the large lobe of the kinase, similar to what was reported in the crystal structure, to others where the J-domain was dislodged from the core of the kinase and swinging free in solution. These simulated dislodged states were experimentally captured by NMR. Modeling of the different conformations revealed no obvious steric interactions of the J-domain with the rest of the RIIβ holoenzyme.

## Introduction

Fibrolamellar Hepatocellular Carcinoma (FLC) is a rare liver cancer usually detected in adolescents and young adults^1^. It does not respond well to chemotherapy, and surgical resection is the primary means of treatment^2,3^. In FLC tumors there is a single, consistent genetic deletion in one copy of chromosome 19^4,5^. This results in the formation of a chimeric gene, *DNAJB1-PRKACA*, which combines the first exon of *DNAJB1*, the heat-shock protein 40 with exons 2 through 10 of *PRKACA*, a catalytic subunit of protein kinase A^4^. There are 3500 statistically significant changes in the transcriptome and proteome when comparing the FLC to the adjacent normal tissue^6^. However, this chimera is the only detected structural alteration of the genome^5^. Expression of the chimera in mouse liver either by recreating the deletion by CRISPR/Cas9, or expression, in trans, by a transposon is sufficient to produce FLC^7^. Thus, it is considered to be the driver for the disease. The chimeric protein encoded by *DNAJB1-PRKACA* comprises of the J-domain of DnaJB1 (the amino-terminal 69 residues), fused to the carboxyl-terminal 336 residues of the PKAcα, the protein encoded by *PRKACA*^4^. The chimeric protein, J-PKAcα, is enzymatically active.

**Figure 1:**
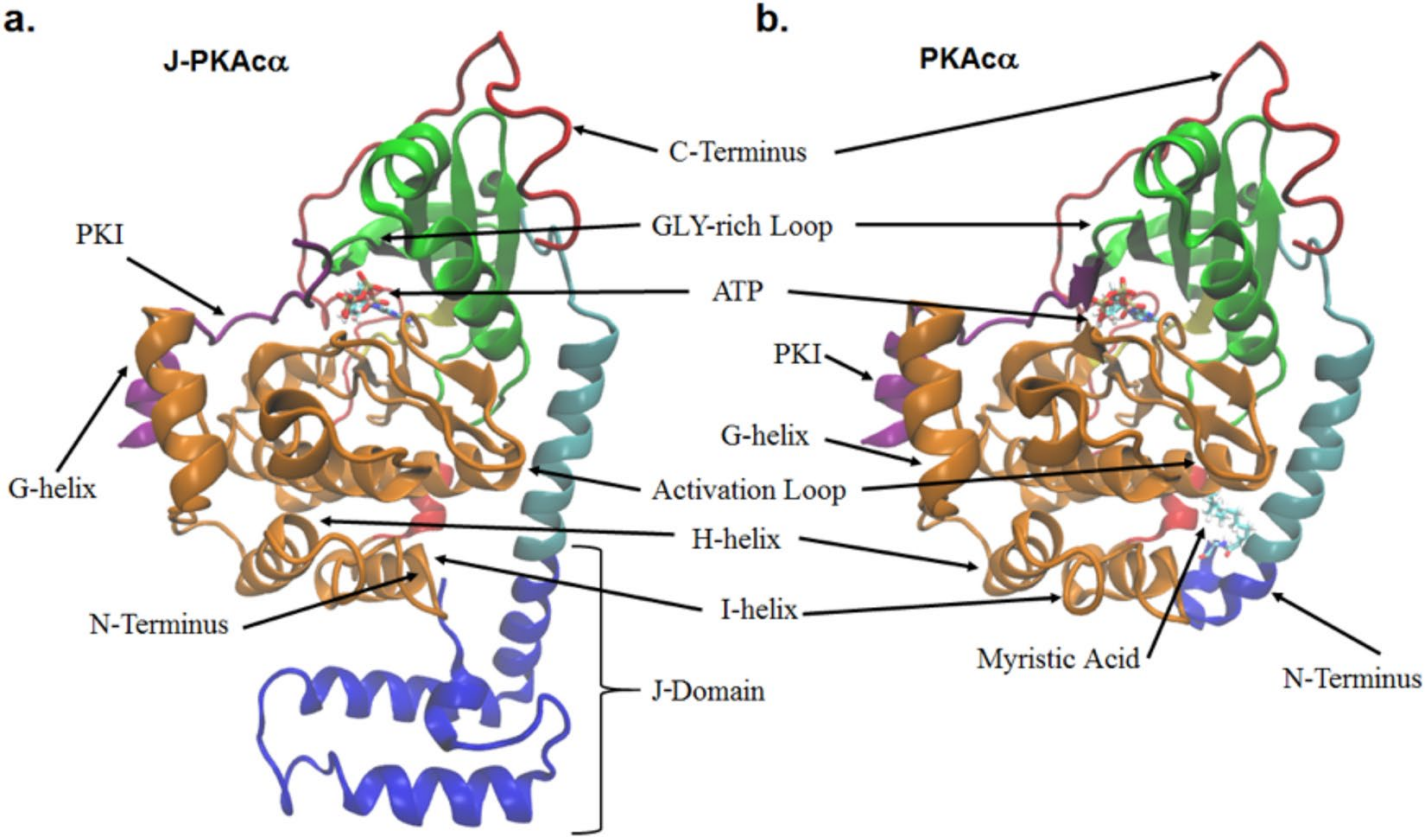
Structures of J-PKAcα chimera and wild-type PKAcα. **(a)** J-PKAcα chimera (PDB ID: 4WB7) with the major domains labeled. **(b)** Wild-type PKAcα (PDB ID: 4DFX). The coloring scheme is as follows: blue = J-domain, Cyan = N-terminal A-helix, Green = Small Lobe, Yellow = Hinge between large and small lobes, Orange = Large Lobe, Red = C-terminal domain, Purple = PKI. ATP, Mg^2+^ ions, and the myristoylation are shown in licorice representation and colored according to atom.

Protein Kinase A (PKA) exists as a heterotetramer comprised of two catalytic (C) subunits, either *PRKACA* (encoding PKAcα), *PRKACB* (PKAcβ), or *PRKACG* (PKAcγ) and a regulatory (R) subunit dimer which comes as either RI (*PRKAR1A* (RIα), *PRKAR1B* (RIβ)) or RII (*PRKAR2A* (RIIα), *PRKAR2B* (RIIβ)) variants. In the holoenzyme, the R-subunits inhibit catalytic activity. A dimerization domain at the N-terminus of each R-subunit is joined by a flexible linker to two tandem cyclic nucleotide binding domains. An inhibitory peptide embedded within the linker of each R-subunit binds in the active site of the C-subunits. When the second messenger 3′,5′-cyclic adenosine monophosphate (cAMP) binds the R-subunits, the C-subunits are able to phosphorylate many downstream targets^8^ involved in a variety of cellular processes^9^. PKAcα is made up of a conserved catalytic core (residues 40–300) consisting of a β-strand rich small lobe and a primarily α-helical large lobe^10,11^. The substrate binding site is mostly in the large lobe while ATP binds in the cleft between the two lobes^12^. The catalytic core is flanked by a 50 amino acid C-terminal segment that wraps around both lobes of the core, while the small lobe is preceded by an N-terminal α-helix termed the A-helix. In the chimeric protein, the lower portion at the amino end of the A-helix is fused to the carboxyl end of the J-domain of DnaJB1 (Fig. 1). In wild-type PKAcα the first exon (residues 1–14) constitutes a myristoylation motif where a myristoyl group on the amino-terminal glycine extends into a hydrophobic cleft in the large lobe. This keeps the A-helix packed closer against the catalytic core of the enzyme compared to non-myristoylated wild-type PKAcα where the first 14 residues are quite flexible^13^. Myristoylation of wild-type PKAcα is thought to be associated with membrane binding of both RII PKA holoenzymes^14^ and allosteric regulation of the ATP active site^15,16^.

A recent crystal structure of the chimeric J-PKAcα protein with ATP and inhibitor peptide (a portion of the cAMP-dependent kinase inhibitor PKI_5-24_) demonstrated that the architecture of the core is conserved compared to wild-type PKAcα^17^. The J-domain extends the A-helix and is tucked beneath the large lobe of PKAcα (Fig. 1). The catalytic core of both PKAcα and J-PKAcα are virtually superimposable in the crystal structures. However, it is possible that the addition of the J-domain could alter conformational dynamics and thus influence substrate binding, enzymatic activity, or regulation^18^. Indeed, the observed B-factors, an indication of the fluctuation of the atomic coordinates relative to their average positions, in the J-domain of the chimera crystal structure were larger than the rest of the protein indicating increased dynamics^17^.

It is unresolved how the physiological properties of the chimera cause FLC. Transformation is not only the consequence of the increased transcription of the *DNAJB1-PRKACA* as a result of expression from the *DNAJB1* promoter since expression of the *DNAJB1-PRKACA* is sufficient for transformation in mouse liver, but expression of the *PRKACA* is not^7^. It could be the result of altered localization of the J-PKAcα fusion protein or interaction with other proteins through the J-domain, effects of the J-domain on the dynamics or specificity of the catalytic core, the absence of myristoylation which could affect either localization or altered kinase dynamics, or the lack of proper regulation of PKA activity by the R-subunits? Any or all of these could contribute to the transformed phenotype. To start exploring these possibilities we performed atomistic molecular dynamics (MD) simulations of both wild-type PKAcα and the J-PKAcα chimera in the ATP-bound, ADP-bound, and Apo states as well as bound to both nucleotide and substrate (ATP and the pseudosubstrate PKI_5-24_) to explore how the addition of the J-domain in the FLC chimera affects the kinase conformational dynamics. We find the J-domain of J-PKAcα samples a wide range of conformations in all substrate/nucleotide binding modes tested. Some conformations show similarity to the crystal structure with the J-domain tucked underneath the large lobe of the kinase, while others show extended conformations that deviated significantly from the crystal structure with the J-domain positioned away from the large lobe. These predictions were confirmed by NMR analysis of the chimeric protein. Structural modeling of different conformational states of the J-PKAcα chimera into a holoenzyme with two RIIβ subunits did not indicate any constraints on the movement of the J-domain.

## Results

To explore the impact of replacing exon 1 of *PRKACA* with exon 1 of *DNAJB1* on the conformational landscape of Protein Kinase A, we performed 1 μs MD simulations on wild-type PKAcα and the J-PKAcα chimera in the ATP-bound, ADP-bound, and Apo states as well as the tertiary ATP and PKI_5-24_ bound states starting from their respective crystal structure. Each simulation rapidly relaxed to a relatively stable state distinct from the starting structure. We quantified the time required for relaxation to this state by calculating the root-mean-squared-deviation (RMSD) of the backbone atoms and comparing it to the average structure over the last 50 ns of simulation time (Supplementary Fig. S1). The structures were aligned using the relatively immobile helices E and F (residues 140 to 160 for helix E and 217 to 233 for helix F with residue numbering from the native PKAcα). The large initial RMSD changes in the J-PKAcα chimera (i.e. 9 Å to 2.5 Å for ATP and PKI-bound) and the non-myristoylated wild-type PKAcα (i.e. 5 Å to 1.5 Å for ATP-bound) indicate a change in the protein conformation, and are primarily the result of the motions occurring at their N-termini: the J-domain and the loop preceding the A-helix for the chimera and wild-type respectively. This can be shown by computing the RMSD of the backbone atoms beginning at residue 15 (70 in J-PKAcα chimera numbering) which omits the N-terminal regions (Supplementary Fig. S1). Omitting these regions, the RMSD values for all proteins change by less than 2 Å during the simulations. All of the simulations shifted away from the crystal structure to a new steady-state following approximately 200 ns. Thus, all subsequent analysis focuses only on snapshots obtained after 200 ns.

**Figure 2:**
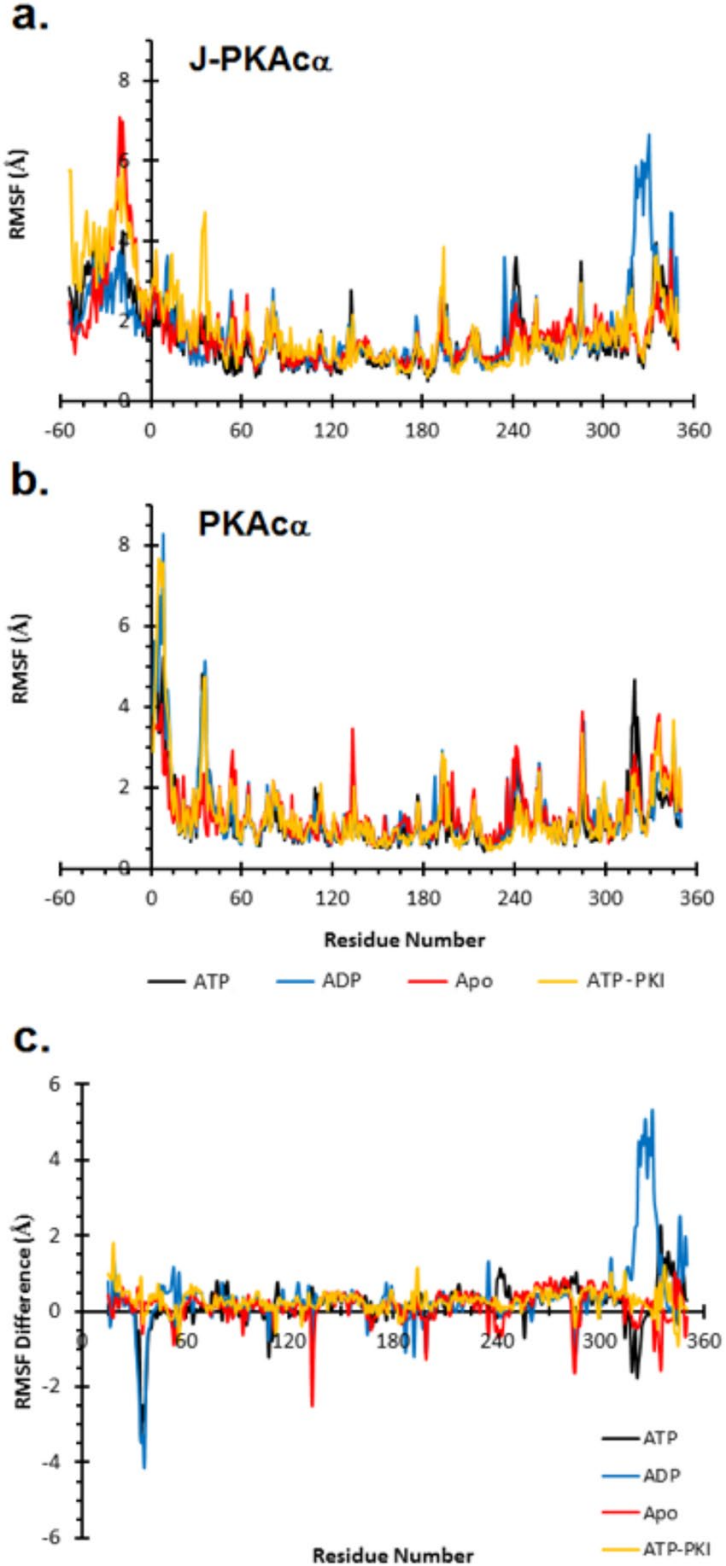
RMSF per residue. RMSF for both J-PKAcα chimera **(a)** and wild-type PKAcα **(b)**. Appended residues in the J-domain of J-PKAcα are numbered negative with residue 15 the first residue that is common to both J-PKAcα chimera and wild-type PKAcα. **(c)** Difference in RMSF (chimera – wild-type) beginning at residue 15 where the residues of the chimera and wild-type are equivalent. Positive numbers indicate greater fluctuations in residues of the chimera while negative numbers indicate greater fluctuation in the wild-type PKAcα.

The degree of local mobility along the kinase was determined by calculating the root-mean-square fluctuation (RMSF) of each residue averaged over the final 800 ns of the simulation and is shown in Fig. 2. For native PKAcα (Fig. 2b), in agreement with previous studies^13,19^, we observed large fluctuations in catalytically important loops such as the Gly-rich loop (residues 50 to 55) and portions of the activation loop (residues 192 to 199). Large fluctuations were also seen in the loop connecting the A-helix to the small lobe (residues 32–37), Lys81 of the B-helix, the loop connecting helices H and I (residues 284 to 287), and residues in the C-terminal tail. Residues of the G-helix (residues 241–245) and Arg133, which are involved in substrate positioning, showed high RMSF values in all simulations except when bound to PKI. The decreased conformational flexibility of those two regions is likely the result of interactions that formed with PKI and stabilized those areas. In simulations of non-myristoylated PKAcα, the N-terminal residues (1–8) were highly dynamic (Supplementary Fig. S2). Overall, the local conformations follow the trend that the Apo state is more dynamic, the intermediate state (nucleotide bound) shows restricted dynamics especially around the nucleotide binding pocket, and the ternary complex is increasingly rigid. This is in agreement with previous simulations and NMR studies of PKAcα which showed the highest degree of fluctuations in the Apo state which decreased as the protein subsequently bound nucleotide and substrate^13,20^.

The chimera showed similar fluctuations to PKAcα in the loop following the A-helix, the Gly-rich loop, the activation loop, the G-helix, and residues in the C-terminal tail (Fig. 2a). Additionally, J-PKAcα demonstrated large fluctuations in the J-domain as a whole. A plot of the differential RMSF between the chimera and native protein highlights the difference in mobility (Fig. 2c). In the ATP- and ADP-bound states, native PKAcα showed increased fluctuations of Asn36 relative to the chimera, which was attenuated in the Apo and ATP-PKI forms. The Apo state of PKAcα had a high amount of fluctuation in Arg133, which was the result of multiple breaking and reforming events of a hydrogen bond between Arg133 and Glu230 which did not occur in the other simulations. The motions of the loop connecting helices H and I are reduced in the chimera compared to PKAcα for the Apo and ADP-bound form, but were not observed in the ATP-bound chimera. This is likely due to the interaction of this loop with the N-terminus of the J-domain. Lys340 and Asn341 form intermittent hydrogen bonds with residues in the J-domain which were not observed in the ATP or ATP-PKI bound forms of the J-PKAcα chimera. The hydrogen bonds result in decreased conformational dynamics of the loop between helices H and I. Finally, there were greater fluctuations in the C-terminal tail of the chimera, especially in the ADP-bound form in which Phe327, which forms the back of the nucleotide binding pocket, was observed to move away from the core of the protein (Supplementary Fig. S3).

**Figure 3:**
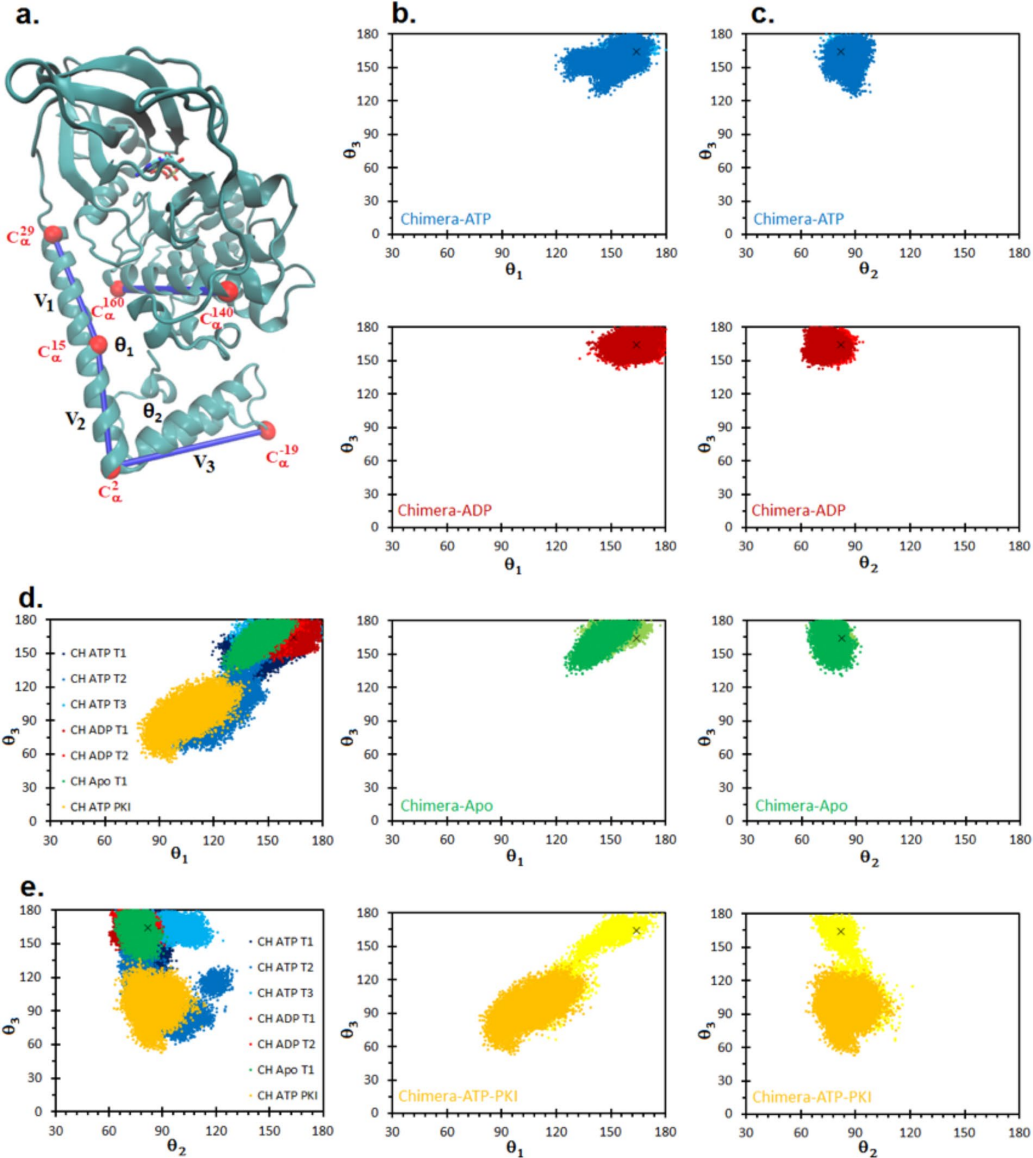
Movement of the J-domain in J-PKAcα chimera. **(a)** Vectors defined to compute the motions of the J-domain as expressed on the crystal structure. The straightness of the A-helix is given by θ_1_, the angle between vectors **ν**_1_ (Cα of Lys29 and Cα of Val15) and **ν**_2_ (Cα of Lys15 and Cα of Pro2). The up and down motion is given by θ_2_, the angle between vectors **ν**_2_ (Cα of Lys15 and Cα of Pro2) and **ν**_3_ (Cα of Pro2 and Cα of Lys^−^19). The azimuthal rotation of the J-domain is given by θ_3_ defined as the dihedral angle between the Cα atoms of Lys29 – Leu160 – Glu140 – Lys^−^19. **(b)** Scatterplots of θ_1_ vs θ_3_ for J-PKAcα chimera for the different binding modes. Lighter colors indicate angles sampled during the first 200 ns of simulation time while darker colors are from the final 800 ns. (**c**) Scatterplots of θ_2_ vs θ_3_ for J-PKAcα chimera for the different binding modes. Lighter colors indicate angles sampled during the first 200 ns of simulation time while darker colors are from the final 800 ns. **(d)** and **(e)** Motions of the J-domain for nine different 1 μs simulations. θ_1_, θ_2_, and θ_3_, are as defined in Fig. 3. **(d)** θ_1_ vs θ_3_ **(e)** θ_2_ vs θ_3_. All data are from the final 800 ns of simulation time. The black ***x*** in the scatterplots corresponds to the crystal structure, PDB ID: 4WB7.

To characterize the motions of the J-domain we defined three vectors (Fig. 3a). We chose the first vector (**ν**_1_) along the A-helix and the second vector (**ν**_2_) at the N-terminal end of the A-helix, which extends into the J-domain. The bending motion of the A-helix is described by the angle between these two vectors (θ_1_). The third vector (**ν**_3_) points from the end of the extended A-helix towards the end of the J-domain. The up/down motion on the J-domain relative to the core of the enzyme is described by the angle between **ν**_2_ and **ν**_3_ (θ_2_). Finally, we define a dihedral angle (θ_3_) given by the positions of the Cα atoms of residues Lys29 – Leu160 – Glu140 – Lys^−^19 that describes the shearing motion between the J-domain and the large lobe.

In the presence of the J-domain there is a kink in the A-helix centered near Arg10 (Arg65 in chimera numbering) that is most pronounced in the ATP-PKI bound form. The bending of this helix, given by θ_1_, is 161° in the crystal structure^17^, and samples values as low as 77° in the simulation (Fig. 3b). The kink is less prominent in the ADP and Apo forms of the chimera, yet both forms still sample values of θ_1_ distinct from the crystal structure (minimum values of θ_1_ = 133° and θ_1_ = 124° respectively). Likewise, for movement of the amino-terminus (θ_2_) the ATP-PKI bound chimera showed the greatest range of motion sampling angles from 62° to 121° (Fig. 3c). The larger values of θ_2_ sampled by ATP-PKI bound chimera correspond to a downward movement of the J-domain away from the large lobe (movement away from the initial starting structure as indicated by the black ***x***). Both movements may be related to the shearing motion (θ_3_) of the J-domain, which also shows the greatest range of motion in the ATP-PKI bound simulation. During the initial 200 ns of the simulation (lighter colors in Fig. 3b,c) the J-domain of ATP-PKI bound chimera transitions from tucked under the large lobe, similar to what is observed in the crystal structure, to a more extended conformation with the N-terminal portion of the J-domain rotated away from the crystal structure conformation. The ATP, ADP, and Apo forms of the chimera also show movement of the J-domain away from its position in the crystal structure, though to a lesser extent than ATP-PKI bound chimera.

**Figure 4:**
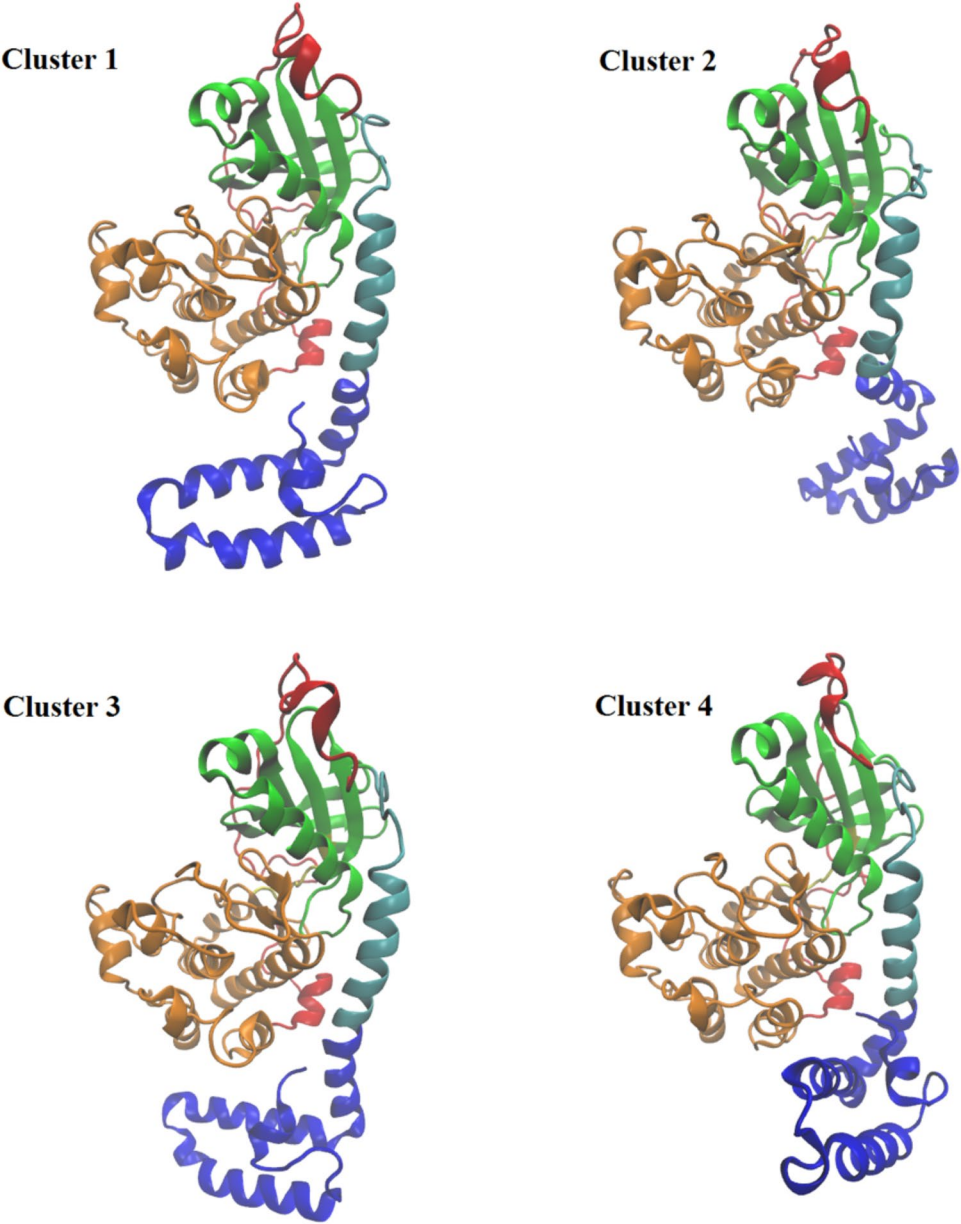
Top four clusters by population from cluster analysis. The coloring scheme is the same as in Fig. 1. Representative structures are the structure with the smallest average RMSD distance to every other member of the cluster. Cluster 1 comprises 62% of all conformations, Cluster 2 comprises 13% of conformations, Cluster 3 comprises 9% of conformations, and Cluster 4 comprises 7% of conformations. The cluster analysis found a total of 21 clusters.

The simulations are consistent with the substrate/nucleotide binding state having an influence on the range of motions of the J-domain (Fig. 3). However, it is conceivable that the 1 μs simulation time was not enough to sample all of the possible conformations of a given binding state. This was tested by performing three more 1 μs simulations, two of the ATP-bound and one ADP-bound chimera. The aggregate data for the J-domain motions from all seven 1 μs simulations is shown in Fig. 4d,e. While the J-domain in the second 1 μs simulation of the ADP-bound chimera (CH ADP T2) sampled conformations similar to the first simulation (CH ADP T1), the additional ATP-bound chimera simulations sampled other conformational states. The second ATP-bound chimera simulation (CH ATP T2) sampled conformations that much resembled the ATP-PKI bound chimera with J-domain extended away from the large domain, as indicated by decreased values of θ_1_ and θ_3_ (minimum value of θ_1_ in CH ATP T1: 119° vs minimum value of θ_1_ in CH ATP T2: 93° and minimum value of θ_3_ in CH ATP T1: 123° vs minimum value of θ_3_ in CH ATP T2: 61°). The third ATP-bound chimera simulation (CH ATP T3) did not show as great a range of conformational space, but did sample larger values of θ_2_ indicating a downward shift of the J-domain relative to the crystal structure.

As the conformational space of the J-domain in the ATP-bound chimera showed variations between the three 1 μs simulations, we attempted to obtain converged results by carrying out a series of ten shorter simulations starting from different initial configurations taken from the three 1 μs simulations. Four conformations were taken from CH ATP T1 (at 200 ns, 466 ns, 733 ns, and 1000 ns), three conformations from CH ATP T2 (at 200 ns, 600 ns, and 1000 ns) and three conformations from CH ATP T3 (at 200 ns, 600 ns, and 1000 ns) and each were extended for 300 ns. The results showed a wide range of states that the J-domain could sample in the ATP-bound chimera, especially in the rotation of the J-domain indicated by θ_3_ values ranging from 24° to 180° (Supplementary Fig. S4).

To further probe the range of conformations that are accessible to the J-domain, and gain insight into the relative populations of the conformations, we performed a clustering analysis based on the RMSD distance of the backbone atoms in each chimera structure. All chimera simulations were combined for a total aggregate time of 10 μs. Using an RMSD cutoff of 5 Å, the chimera simulations separated into 21 clusters with the first four clusters (Fig. 4) accounting for 91% of the total conformations (Supplementary Fig. S5). Pooling the wild type simulations and performing a cluster analysis with the same parameters resulted in a total of 2 clusters with the vast majority (greater than 99%) populating the first cluster indicating a larger conformational ensemble of structures in J-PKAcα compared to the wild type protein. The first J-PKAcα cluster comprises the great majority of conformations accounting for 62% of all structures. A variety of conformations for the J-domain was observed in the top four clusters (Fig. 4). The first cluster shows similarities to the crystal structure (compare to Fig. 1) with similar angles for the J-domain (Table 1). Cluster 2 shows the kink near Arg10 in the A-helix resulting in a decreased value of θ_1_ compared to the crystal structure (119° vs 164°). This is also accompanied by a downward motion (increase in θ_2_ compared to the crystal structure) and a rotation (decrease in θ_3_ compared to the crystal structure) of the J-domain. Cluster 3 shows the J-domain tucked underneath the large lobe, similar to the crystal structure, but there is also a kink near Arg10 similar to cluster 2. Finally, cluster 4 shows a rotation of the J-domain in the opposite direction from the rotation in cluster 2.

**Table 1:** Angles of the chimera J-domain. The angles are defined in Fig. 3. The top four clusters from cluster analysis are compared to the crystal structure PDB ID: 4WB7.

	θ_1_	θ_2_	θ_3_
Crystal Structure	164°	82°	164°
Cluster 1	169°	89°	166°
Cluster 2	119°	98°	101°
Cluster 3	153°	88°	145°
Cluster 4	157°	75°	163°

We examined the frequency of transitions between the different clusters. At each time point we determined if the initial conformation transitioned to a different state (Supplementary Fig. S6) or remained in the same state (Supplementary Fig. S6). The frequency of transitions is given by a color code. The time steps where the state did not change were much more frequent than transitions to different states. In general, the transitions between clusters are symmetrical, i.e. cluster 1 is most likely to transition to cluster 4 and cluster 4 is most likely to transition to cluster 1. A number of transitions were not preferred. For example, in the 10 μs of aggregate simulation time, there was only one transition between the two most populated clusters, cluster 1 and cluster 2. Both clusters, much more frequently transition to cluster 3, which then shows a moderate number of transitions back to both clusters 1 and 2.

**Figure 5:**
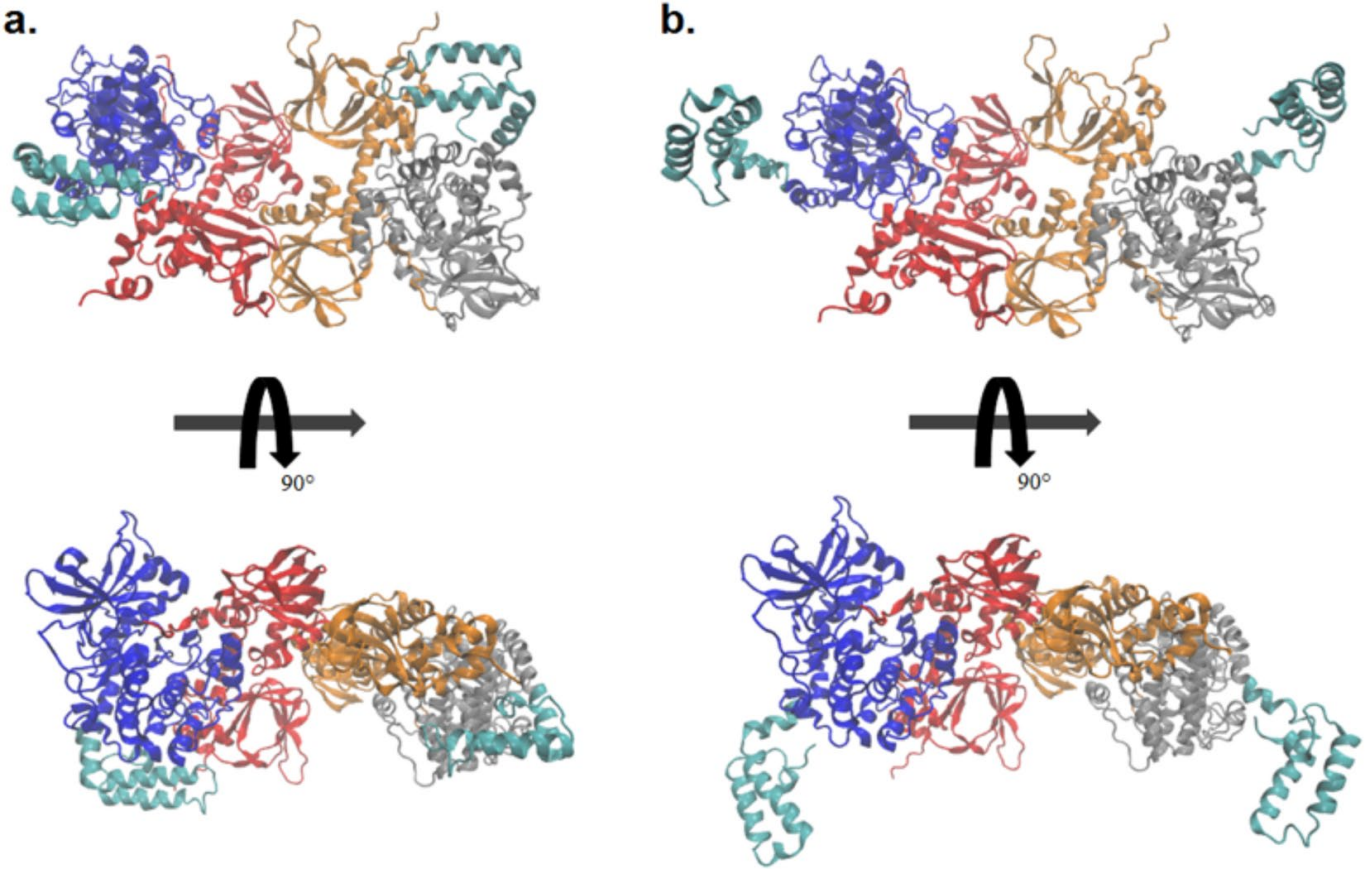
Top clusters from cluster analysis modeled into the RIIβ holoenzyme. **(a)** Cluster 1 in a ‘J-in’ state and **(b)** Cluster 2 in a ‘J-out’ state from Fig. 4. The two RIIβ subunits are colored in orange and red, the two C-subunits without the J-domains are colored blue and gray, and the J-domain is colored in cyan.

All of these studies are consistent with the J-domain being freely mobile and able to explore a variety of conformations in the isolated chimera kinase. We wanted to examine if the same mobility of movement was possible for the chimeric kinase in the context of the holoenzyme. Using a crystal structure of PKAcα bound to the R-subunit(RIIβ)^21^, the J-domain from the top two clusters of the cluster analysis were modeled into the holoenzyme by aligning the backbone atoms of helices E and F and with the wild type PKAcα contained in the holoenzyme. In the holoenzyme the RIIβ subunits do not demonstrate any steric hindrance to the motions of the J-domain either when it is in a ‘J-in’ state (as in cluster 1) tucked underneath the core of the kinase or when in a ‘J-out’ state (as in cluster 2) when the domain is positioned far away from the catalytic core (Fig. 5). This would suggest that while in the holoenzyme the J-domain can be similarly mobile as in the isolated C-subunit. As a caveat, the first 103 N-terminal residues of RIIβ structure, containing the AKAP binding domain, were not resolved in the crystal structure. Thus, it is possible that this N-terminal domain is located such that it would interact with the J-domain and alter its conformational dynamics.

**Figure 6:**
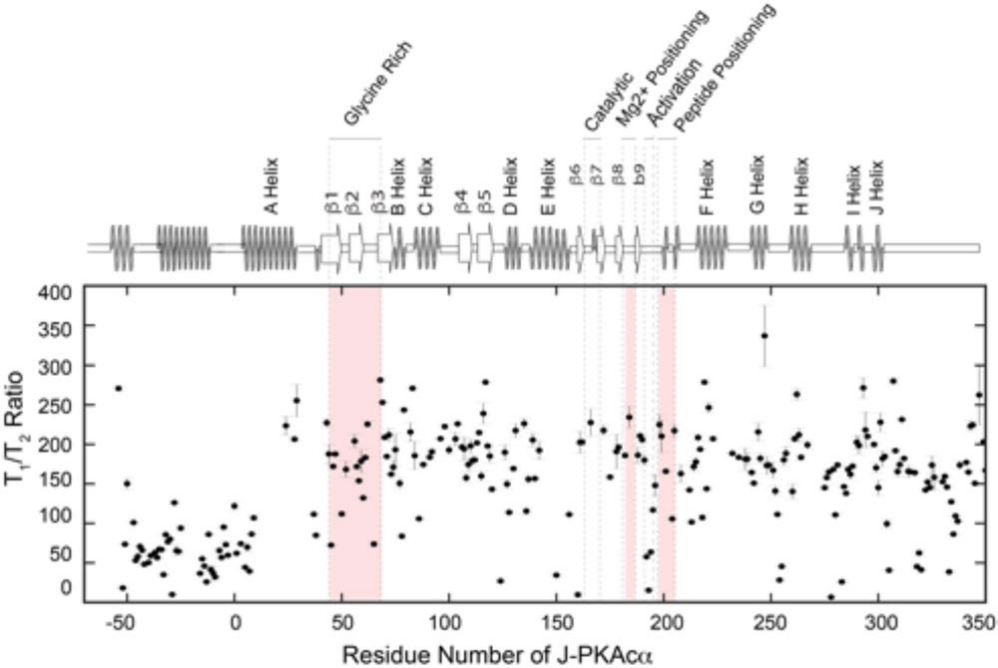
Residue-specific T_1_/T_2_ ratio of NMR relaxation of J-PKAcα. Experimental values of residues in J-domain are much smaller than the values of other domains, suggesting a significantly higher flexibility of the appendage.

The MD simulation results indicate that the J-domain of the chimeric kinase is freely mobile. To test experimentally the conformational dynamics of the J-domain, we used NMR nuclear spin relaxation measurements. The resonance assignments for the catalytic core of the J-PKAcα fingerprint were transferred from the previous chemical shift assignments of the wild-type PKAcα amide resonances^22^. To assign the J-domain, we recombinantly synthesized and purified a DnaJB1 peptide from residue 1 to 76. The chemical shift assignments of the DnaJB1 peptide were then compared to the corresponding resonances of the J-domain in the full-length J-PKAcα. Ambiguous or overlapped peaks were assigned using the TROSY-version^23^ of triple-resonance experiments^24^ with U-^2^H,^15^N,^13^C labeled J-PKAcα. The direct correspondence between the resonances of the isolated constructs and the full-length J-PKAcα indicates that this domain folds independently^25^. To assess the global motions of J-PKAcα, we measured both the ^15^N longitudinal (T_1_) and transverse (T_2_) relaxation times^26^ (Supplementary Fig. S7). From the analysis of the relaxation times of the core of the enzyme (*i.e*., without the J-domain), the ternary form of the J-PKAcα chimera reorients faster (22.9 ns) than the ternary form of wild-type PKAcα (24.0 ns). Given that the mass of the J-PKAcα chimera is larger than that of the wild-type PKAcα, one might expect the chimera to have a slower rotational correlation time in NMR. In fact, a theoretical calculation using HYDRONMR^27^ estimates a correlation time of 29.4 ns for the wild-type enzyme (PDB: 1ATP); while it predicts 37.4 ns for the J-PKAcα chimera (PDB: 4WB7). Since HYDRONMR assumes a rigid body reorientation of the molecule, the experimental observation of a faster rotational correlation time for the J-PKAcα chimera is consistent with the J-domain having a high degree of flexibility, in agreement with the MD results. Importantly, the plots of the T_1_/T_2_ versus residues reveal two distinct regions for J-PKAcα (Fig. 6): the core of the enzyme, with T_1_/T_2_ values averaging around 200, and the N-terminal region encompassing the J-domain with an average T_1_/T_2_ of 55. These data suggest that the protein does not tumble as a rigid body; rather the catalytic core is disjointed from the J-domain, (residues −69 to −1) which undergoes faster global reorientation.

## Discussion

There is compelling evidence that the J-PKAcα chimera is the main driver for the development of FLC^4–6^. There are a number of possible explanations for how expression of this chimera could lead to transformation. One possibility is that the *DNAJB1* promoter, which drives the chimera, may be expressing more of the C-subunit, and this is sufficient for transformation. A second possibility is that the loss of the amino-terminal myristoylation motif, affects the catalytic rate or localization of PKAcα. A third possibility is that the presence of the J-domain at the amino terminus alters the subcellular localization or binding partners of the C-subunit and/or the PKA holoenzymes. A fourth possibility is that the addition of the J-domain at the amino terminus affects the dynamics and thereby potentially the specificity of the kinase for nucleotide or substrate. A final possibility is the J-domain alters the finely tuned regulation of the PKA holoenzyme. We favor the hypothesis that the addition of the J-domain in some way alters the kinase dynamics, activity, regulation, or localization since the cancer has never been reported with just loss of the amino terminus, just loss of myristoylation on the second glycine, nor with the addition of any other amino terminus. Here, we have focused attention on the dynamic effects of adding the J-domain to the amino-terminal end of PKAcα, the C-subunit of protein kinase A.

In the crystal structure of the RIIβ wild-type holoenzyme the N-terminus of the C-subunit, where the J-domain addition occurs, is positioned away from the major symmetrical interface between the C- and R-subunits in the holoenzyme^21^, and the presence of the J-domain does not prevent formation of the holoenzymes. Thus, rather than affecting the interactions with the regulatory subunits, it is possible that addition of the J-domain alters the conformational landscape of the chimera and the resulting holoenzymes. The higher B-factors in the J-domain^17^ implied a large degree of conformational flexibility. Both simulations and experiment have shown that point mutations in PKAcα far from the active site can affect kinase activity allosterically though decoupling of dynamics^28,29^, implying that motions of the J-domain could influence the allosteric network in J-PKAcα and thereby alter its activity. In this work, we performed several microseconds of MD simulations exploring the conformational states adopted by the kinase and J-domains. We found that a primary conformation, accounting for 62% of all structures sampled by chimera MD trajectories, resembled the crystal structure where the J-domain was tucked underneath the large lobe of the kinase. Nonetheless, we also found an ensemble of chimera conformations in which the J-domain was dislodged from the core of the enzyme allowing it to swing freely in solution. These states were not captured by the X-ray crystal structure, but were confirmed using NMR. The flexible conformations appear to be independent of the nucleotide/substrate binding mode as they were observed in ATP, ADP, Apo, and ATP-PKI bound states. Focusing on the ATP-bound state, we showed that 1 μs of simulation time was not sufficient to obtained a converged ensemble of J-domain configurations as different 1 μs simulations showed a wide variability in the J-domain motions (Fig. 3). Because the conserved core of the wild-type enzyme and the chimeric fusion show little structural differences and the canonical function is not affected by the J-domain appendix, the presence of alternate conformations may constitute a way to target the chimera selectively. This opens up the possibility to develop novel small molecule inhibitors directed at the region of the fusion. However, as our results show, care must be taken when designing inhibitors so as to encompass the wide range of motion of the J-domain, and in particular, the A-helix where the fusion occurs.

## Materials and Methods

### Molecular Dynamics

MD simulations were performed to observe the structural dynamics of J-PKAcα chimera and determine how they differ from wild-type PKAcα. Each protein (wild-type and chimera) was simulated in the ATP-bound, ADP-bound, and Apo forms as well as the tertiary state bound to both ATP and pseudosubstrate PKI_5-24_. Each simulation was built from the crystal structure, PDB ID: 4DFX^15^ in which the protein is in complex with the ATP mimic AMP-PNP, 2 Mg^2+^ ions and the 20 residue peptide SP20. SP20 corresponds to the endogenous PKA inhibitor PKI_5-24_ with two mutations (N20A and A21S) which convert PKI_5-24_ from inhibitor to substrate. The crystal structure of PKAcα also includes the myristoylation at the N-terminus as well as the mutation K7C. In all wild-type PKAcα simulations the cysteine at position 7 was mutated back to a lysine using the Schrodinger Maestro software suite (Maestro 10 (2016) Schrödinger, Inc., Portland, OR). AMP-PNP was either mutated to ATP or ADP or removed for the Apo state. 2 Mg^2+^ ions were retained for the ATP simulations, 1 for the ADP simulations, and removed for the Apo simulations. SP20 was mutated back to PKI_5-24_ in simulations of the tertiary state. The chimeric J-PKAcα systems were built using chain A of the crystal structure, PBD ID: 4WB7^17^ in which the protein is bound to ATP, several Zn^2+^ ions, and PKI_5-24_. As with the wild-type PKAcα simulations, J-PKAcα chimera was simulated in the ATP, ADP, and Apo states as well as the tertiary state with ATP and PKI_5-24_ bound. Zn^2+^ ions associated with nucleotide were converted to Mg^2+^ ions, with other Zn^2+^ ions removed. All structures were phosphorylated at S139, T197 and S338 (amino acid numbers for the native PKAcα or S194, T252 and S393 in J-PKAcα chimera numbering). Structures were processed using the Protein Preparation Wizard in Maestro (Maestro 10 (2016) Schrödinger, Inc., Portland, OR), solvated in a rectangular box with SPC waters, and sodium or chloride ions were added for electrostatic neutrality. Simulations were performed using the Desmond MD Package^30^ using the OPLS3 force field^31^. Each system was subject to energy minimization using the steepest decent method. An initial 100 ps Brownian Dynamics simulation at constant volume and a temperature of 10 K with heavy atoms constrained was performed. Subsequent equilibration included a 12 ps simulation at constant volume and at 10 K with heavy atoms restrained, followed by a 12 ps simulation at constant pressure with heavy atoms restrained, and finally a heating simulation in which the restraints were gradually relaxed and the system heated to 300 K over 24 ps. Production simulations were performed for 1 μs or 300 ns with system snapshots saved every 50 ps.

### Clustering of Conformations

All simulations were aggregated to generate distinct clusters of conformational states. Helices E (Residues 140–160) and F (Residues 217–233) where used to align all trajectories to the initial crystal structure. Analysis was performed on the protein backbone atoms first using the GROMACS analysis tool ‘gmx rms’ to compute the root-mean-square-deviation between all structures in the trajectory. The GROMACS utility ‘gmx cluster’ was then used to cluster the structures using a cutoff of 5 Å and the gromos clustering methodology^32^. Representative structures were taken as the structure with the smallest average RMSD distance to every other member of the cluster.

### Protein expression, purification, and NMR sample preparations

High-level expression of J-PKAcα in E. coli was achieved by the construction of pET28a(+) vector that contained the protein gene subcloned prior to a phage T7 RNA polymerase promoter. A TEV protease cleavage sequence was engineered between the Histidine (His) tag sequence and target gene. Uniformly ^2^H,^15^N-labeled J-PKAcα was expressed using the *E. coli* BL-21(DE3) bacterial strain in M9 medium containing ^15^NH_4_Cl as the sole nitrogen source and transferred to an 80% ^2^H containing M9 media. His-tagged J-PKAcα was purified using TALON Metal Affinity Resin (Clontech). The His-tag was removed using a modified TEV protease protocol^33^ (Supplementary Fig. S8) followed by an additional purification step using FPLC with an ion exchange column (HiTrap® Q-SP). The wild-type PKAcα was expressed in the same media and purified as described previously^34^. The 69 amino acid sequence corresponding to the *DNAJB1* fragment was cloned into pET-28a(+) vector. An affinity His-tag was engineered after the sequence separated by a thrombin cleavage site. *E. coli* strain BL-21 was used as a bacterial host for the overexpression. The purified fusion protein was cleaved using thrombin protease, diluting 500 units of enzyme in 0.5 ml into cold PBS buffer (140 mM NaCl; 2.7 mM KCl; 10 mM Na_2_HPO_4_; 1.8 mM KH_2_PO_4_; pH = 7.3). The cleavage reaction was monitored by SDS-PAGE. Upon completion the thrombin was inactivated by adding of 1 mM PMSF. The NMR samples of ternary complexes were prepared using ^2^H,^15^N labeled J-PKAcα and wild-type PKAcα in a 2:1 ratio with PKI_5-24_ and in the presence of 12 mM of ATPγN. The proteins were solubilized in 250 μl of 95% H_2_O and 5% D_2_O buffer solution, containing 20 mM potassium phosphate, 180 mM KCl, 10 mM MgCl_2_ (pH = 6.5). The final sample concentrations were 180 μM and 250 μM for the chimera and the wild-type kinase complexes, respectively. Uniformly ^2^H,^15^N,^13^C labeled DnaJB1 was prepared in 250 μl of 95% H_2_O and 5% D_2_O in 20 mM potassium phosphate buffer with 180 mM KCl and 10 mM MgCl_2_ (pH 7.0) to a final concentration of 200 μM.

### NMR Spectroscopy

NMR experiments were carried out on 850 and 900 MHz Bruker spectrometers equipped with a cryogenic probe. The temperature was held constant at 300 K. Resonance assignments of the amide fingerprint of J-PKAcα was carried out by transferring the previous assignments from wild-type PKAcα. The DnaJB1 amide fingerprint and side chains were assigned using a combination of 3D CBCACONH^35^ and HNCACB^36^ experiments. Longitudinal (T_1_) and transverse (T_2_) relaxation times were evaluated by monitoring the signal intensity decay of the amide resonances in the series of 2D spectra using the pulse sequences described by Zhu *et al*.^37^. The spectra were recorded in an interleaved manner for both T_1_ and T_2_. The spectral dimensions were 3466 Hz (F1) and 14423 Hz (F2). For both T_1_ and T_2_, the relaxation decay was sampled for 8 different delays. For each T_1_ value, the order of the relaxation delay was randomized (T_1_ delays: 0, 10, 100, 300, 500, 700, 900, 1200 and 1700 ms) with 32 scans per FID. For T_2_, the relaxation delays were 8.1, 16.2, 24.3, 32.4, 40.5, 48.6, 56.7, and 64.8 ms with 96 scans per FID. Least-squares fitting of the decay curves was carried out with the downhill simplex algorithm implemented into SPARKY (Goddard and Kneller). Backbone assignments of J-PKAcα was achieved by overlay the resonances of the previously assigned wild-type PKAcα and transferring the resonance assignments of DnaJB1. All NMR data were processed using the NMRPipe suite of programs^38^.

## Electronic supplementary material

Supplementary Information
