# Supplementary material for: Conformational Landscape of the PRKACA-DNAJB1 Chimeric Kinase, the Driver for Fibrolamellar Hepatocellular Carcinoma
Source: Sci Rep. 2018 Jan 15;8:720. doi: 10.1038/s41598-017-18956-w (PMC5768683; doi:10.1038/s41598-017-18956-w)
Supplement: Supplementary file 1 — Supplementary Information [file 41598_2017_18956_MOESM1_ESM.doc]

**Supplemental Material**

**Conformational Landscape of the *PRKACA-DNAJB1* Chimeric Kinase, the Driver for Fibrolamellar Hepatocellular Carcinoma**

Michael D. Tomasini^1^, Yingjie Wang^2,3^, Adak Karamafrooz^3^, Geoffrey Li^2^, Thijs Beuming^5^ Jiali Gao^2,6^, Susan S. Taylor^4,7^, Gianluigi Veglia^2,3^, and Sanford M. Simon^1,*^

**^1^**Laboratory of Cellular Biophysics, The Rockefeller University, 1230 York Avenue, New York, NY, 10065, USA.

^2^Department of Chemistry, University of Minnesota, Minneapolis, MN 55455, USA.

^3^Department of Biochemistry, Molecular Biology, and Biophysics. University of Minnesota, Minneapolis, MN 55455, USA.

^4^Department of Pharmacology, University of California at San Diego, CA 92093, USA.

**^5^**Schrödinger Inc., 120 West 45^th^ Street, New York, NY, 10036 USA.

^6^Theoretical Chemistry Institute, State Key Laboratory of Theoretical and Computational Chemistry, Jilin University, Changchun, Jilin Province 130028, People’s Republic of China.

^7^Department of Chemistry and Biochemistry, University of California at San Diego, CA 92093, USA.

**^*^**Corresponding Author: Email: simon@rockefeller.edu


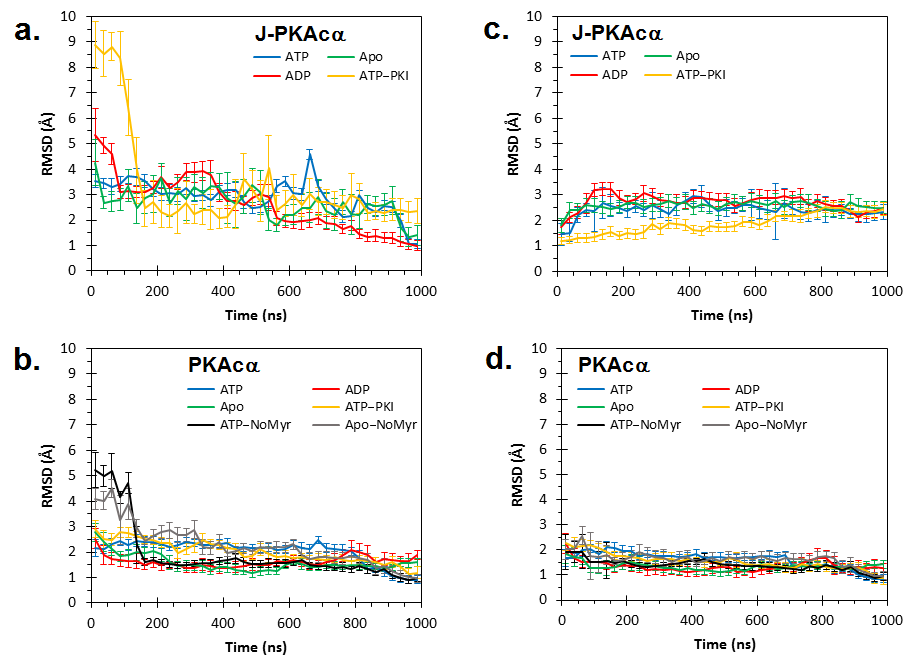


**Supplemental Figure S1.** RMSD to the average structure of the last 50 ns of simulations time. **(a)** J-PKAcα chimera and **(b)** wild-type PKAcα RMSD calculated using the full length proteins. **(c)** J-PKAcα chimera and **(d)** wild-type PKAcα RMSD in which the first 69 residues for J-PKAcα and the first 14 residues for wild-type PKAcα were not included in the calculation. Points are averages over 25 ns intervals, and error bars show the standard deviation over the interval.


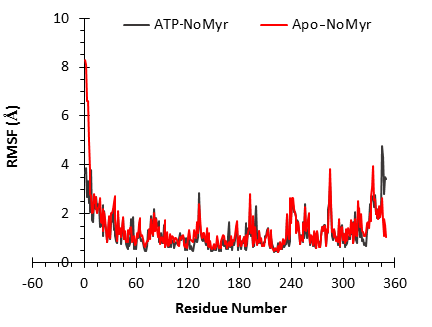


**Supplementary Figure S2.**  RMSF for non-myristoylated wild-type PKAcα. ATP-NoMyr = ATP-bound non-myristoylated PKAcα, Apo-NoMyr = unbound non-myristoylated PKAcα.


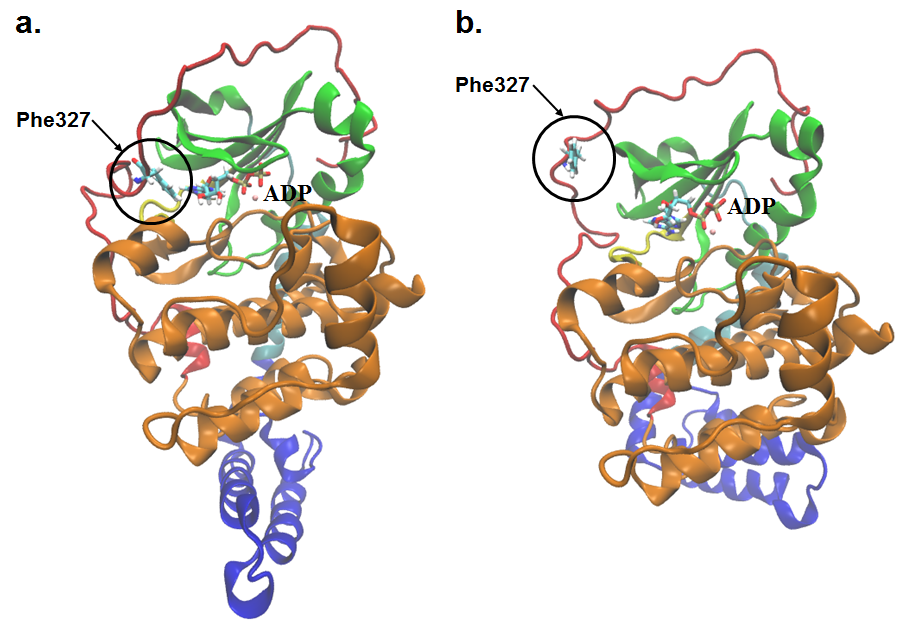


**Supplementary Figure S3.** Position of Phe327 in ADP-bound J-PKAcα simulations. Initial **(a)** and final **(b)** structures of ADP-bound J-PKAcα chimera. The initial structure has Phe327 forming the back of the ADP-binding pocket. Over the course of the 1 μs simulation, Phe327 moves away from its initial position exposing the binding pocket to solvent.


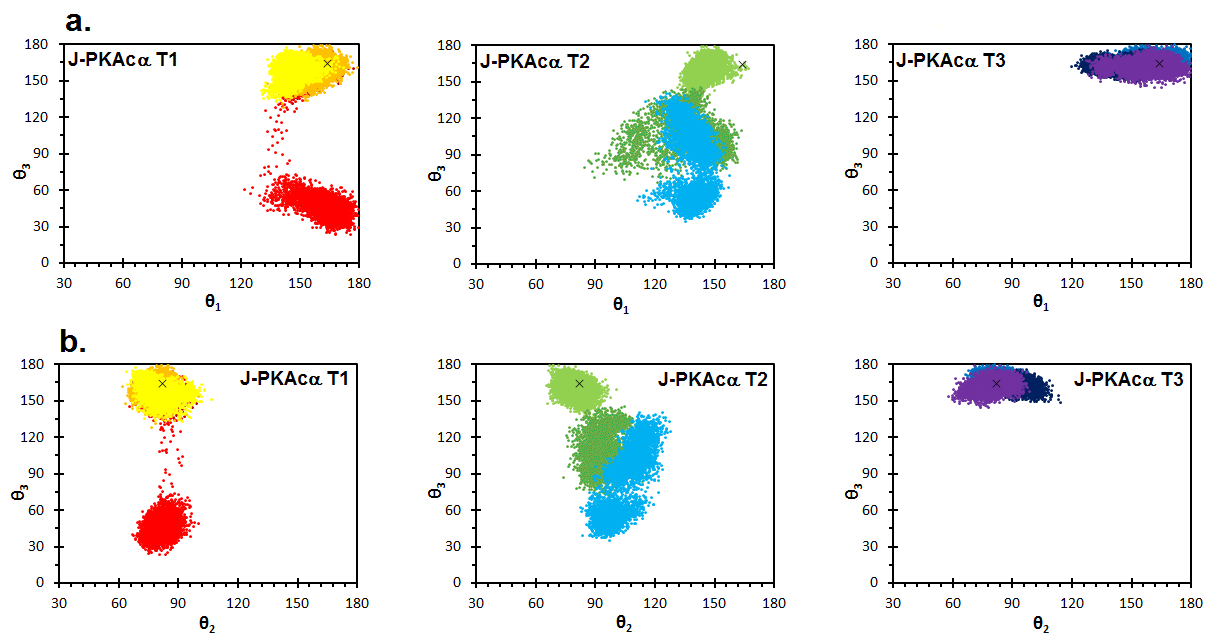


**Supplementary Figure S4.** Movement of the J-domain in ATP-bound J-PKAcα with angles defined according to Fig. 4. Each simulation ran for 300 ns and started from a snapshot of a longer 1 μs run. Four simulations were run taking structures from the first 1 μs run (J-PKAcα T1) at 200 ns, 466 ns, 733 ns, and 1000 ns. Three simulations were run taking structures from the second 1 μs run (J-PKAcα T2) at 200 ns, 600 ns and 1000 ns. Finally, three simulations were run taking structures from the third 1 μs run (J-PKAcα T3) at 200 ns, 600 ns, and 1000 ns. **(a)** Scatterplots of θ_1_ vs θ_3_ for ATP-bound J-PKAcα. **(b)** Scatterplots of θ_2_ vs θ_3_ for ATP-bound J-PKAcα. The black **x** in the scatterplots indicates the angles of the crystal structure, PDB ID: 4WB7.

**
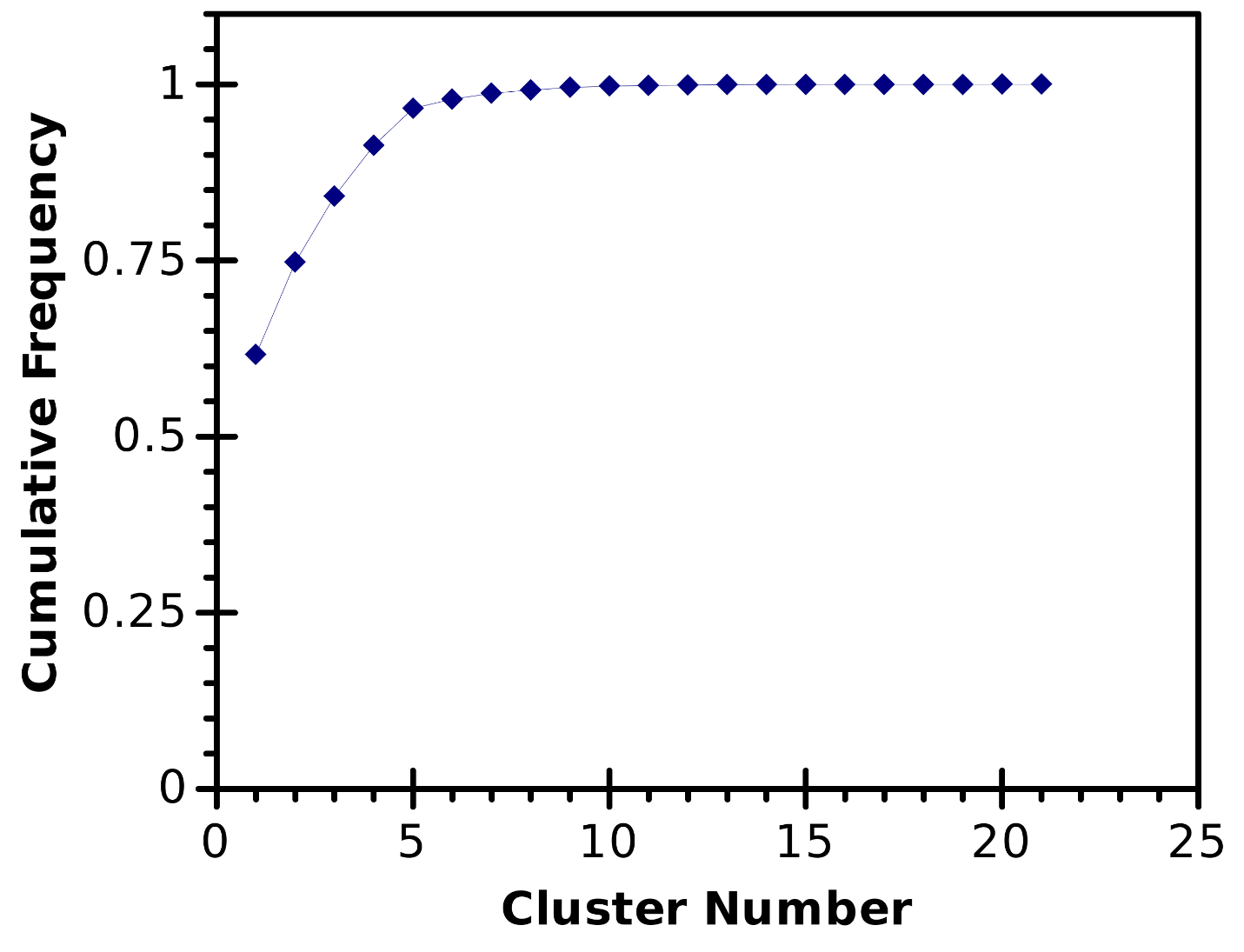
**

**Supplementary Figure S5.** Cumulative frequency of each cluster from the cluster analysis. Cluster analysis was performed with an RMSD cutoff of 5 Å. The first four clusters account for greater than 90% of all chimera conformations.


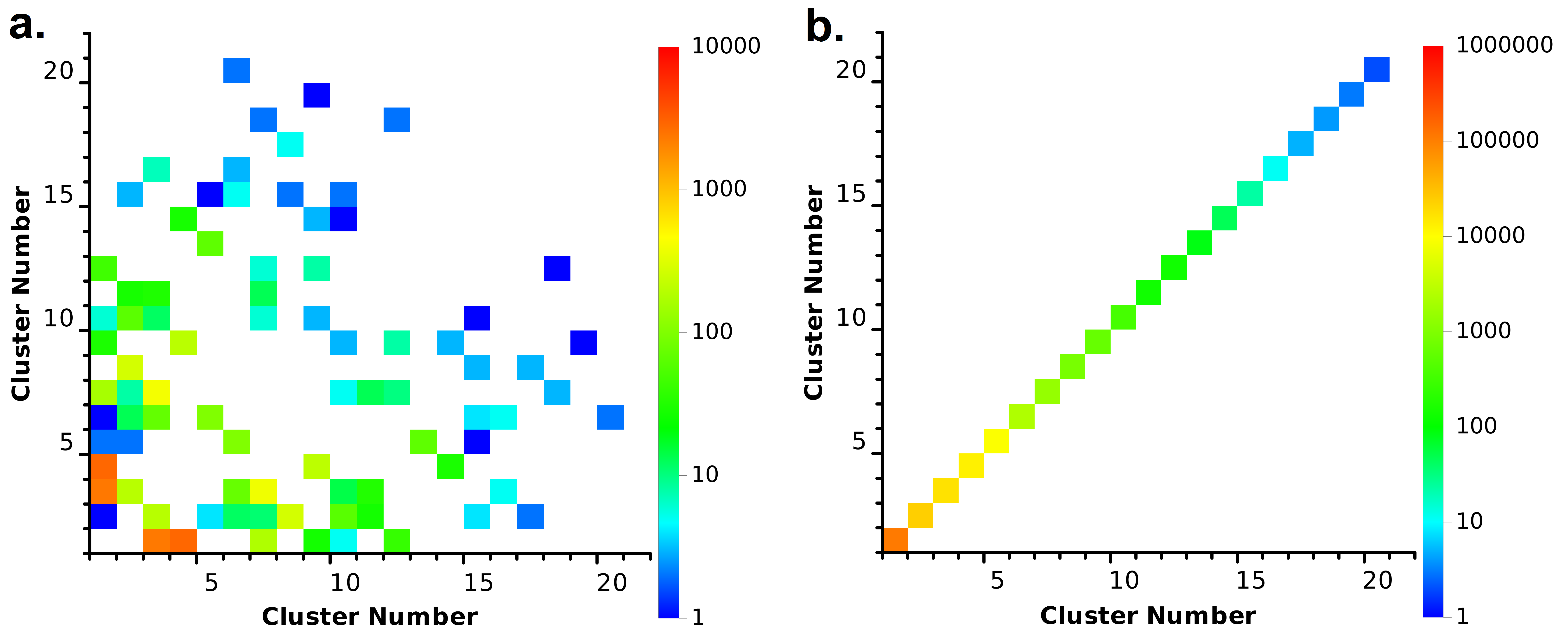


**Supplementary Figure S6.** Cluster analysis of all chimera conformations. **(a)** Number of transitions between pairs of clusters with the initial conformation on the x-axis and the transition conformation on the y-axis **(b)** Number instances when a conformation does not transition to a new cluster.


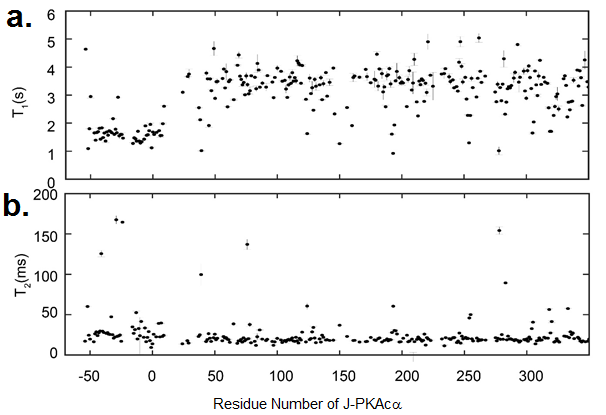


**Supplementary Figure S7.** Residue-specific relaxation values for the J-PKAcα chimera. **(a)** T_1_ and T_2_ **(b)** values for J-PKAcα residues in J-domain appendix. Experiment T1 values are much smaller than the values of other domains, suggesting the exceptional flexibility of the J-domain.


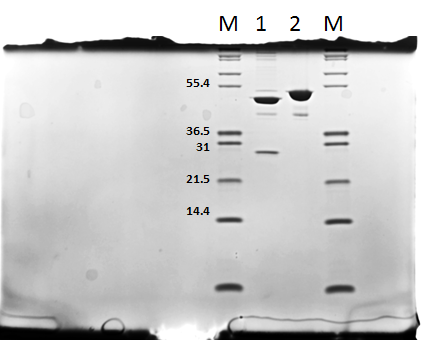


**Supplementary Figure S8.** Cleavage reaction in His-tagged J-PKAcα detected by 12% SDS-Page. Lane 1: J-PKAcα sample after TEV cleavage reaction. Lane 2: J-PKAcα prior to cleavage. Protein Marker: Mark12^TM^ Unstained Standard from ThermoFisher Scientific.
